# A Semi-implicit Treatment of Porous Media in Steady-State CFD

**DOI:** 10.1007/s11242-016-0657-3

**Published:** 2016-02-26

**Authors:** Andreas Domaingo, Daniel Langmayr, Bence Somogyi, Raimund Almbauer

**Affiliations:** VIRTUAL VEHICLE Research Center, Inffeldgasse 21a, 8010 Graz, Austria; ANDRITZ HYDRO GmbH, Dr.-Karl-Widdmann-Strasse 5, 8160 Weiz, Austria; KTM AG, Stallhofnerstrasse 3, 5230 Mattighofen, Austria; Graz University of Technology, Inffeldgasse 25b, 8010 Graz, Austria

**Keywords:** Darcy–Forchheimer, Implicit source term, Coarse grid CFD

## Abstract

There are many situations in computational fluid dynamics which require the definition of source terms in the Navier–Stokes equations. These source terms not only allow to model the physics of interest but also have a strong impact on the reliability, stability, and convergence of the numerics involved. Therefore, sophisticated numerical approaches exist for the description of such source terms. In this paper, we focus on the source terms present in the Navier–Stokes or Euler equations due to porous media—in particular the Darcy–Forchheimer equation. We introduce a method for the numerical treatment of the source term which is independent of the spatial discretization and based on linearization. In this description, the source term is treated in a fully implicit way whereas the other flow variables can be computed in an implicit or explicit manner. This leads to a more robust description in comparison with a fully explicit approach. The method is well suited to be combined with coarse-grid-CFD on Cartesian grids, which makes it especially favorable for accelerated solution of coupled 1D–3D problems. To demonstrate the applicability and robustness of the proposed method, a proof-of-concept example in 1D, as well as more complex examples in 2D and 3D, is presented.

## Introduction

Source terms are frequently encountered in computational fluid dynamics. They are necessary in situations, where processes affect the flow which are not resolved by the Navier–Stokes equations or the applied numerical grid. Examples of such processes are chemical reactions, turbulence, geometry simplifications in porous media, body force terms like buoyancy, etc. Such source terms not only affect the physics of the flow solution, but also have a large effect on the applied numerical scheme in terms of reliability, stability, and convergence behavior. One reason for an undesirable blow up due to source terms is that in the course of integration, the solution may come into regimes where the source terms exhibit large unrealistic values dominating the actual physics. Therefore, special care has to be taken with regard to source terms in order to guarantee solver robustness.

Numerous different approaches have been proposed to diminish the disadvantageous effects of source terms. In Yu and Chang (1997), a conservative interpolation of source terms in space–time is presented. A robust solution for conservation laws with source terms by means of a wave-propagation algorithm is discussed in LeVeque (1998). A discretization of the source terms by taking the natural upwind direction into account is considered in Vázquez-Cendón (1999) for the shallow water equations. A technique for balancing flux gradients and source terms is investigated in Hubbard and Garcia-Navarrot (2000). Implicit integration schemes of different order are evaluated in Mulder (1985) for the one-dimensional Euler equation. Other methods concentrate on solution-limited time stepping (Lian et al. 2009, 2010). In the latter method, the CFL number is reduced locally, to ensure the change in each cell to be below a specified tolerance thereby enhancing code reliability. Usually, as an initial step, the choice of implicit or explicit treatment has to be made (Piscaglia et al. 2009). This can be done based on the Jacobian of the source term or on the basis of individual terms (Merci et al. 2000). Generally speaking, positive source Jacobian eigenvalues are better handled by explicit algorithms, whereas negative eigenvalues improve the convergence of implicit approaches.

In this paper, we present an approach to efficiently model the influence of porous media resolved by source terms for steady-state CFD calculations. This is a common approach for investigations in which the porous medium cannot be fully resolved by the numerical grid (Hayes et al. 2008; Aslan et al. 2014; Hörmann et al. 2005). A well-known application is, e.g., the air flow in a vehicles underhood compartment or in HVAC ducts in the presence of heat exchangers (Janssen et al. 2015), where only the influence of the heat exchanger on the overall pressure loss of the system is of interest. In this case, the resistance, i.e., the local pressure loss, can be expressed by a suitable source term.

Our emphasis is on methods, in which the solution is found by quasi-time relaxation to the equilibrium state. Such a method has been proposed by the authors in Langmayr et al. (2013) for a calibrated coarse grid method (CCGM). The main goal of this approach is to reproduce the average results of a CFD simulation on a very fine grid with an under-resolved solution of the Euler or Navier–Stokes equations. This is established via appropriate averaging and source terms. The relaxation is controlled by a global relaxation time step, which is small enough to ensure a suitably small CFL number in the entire computational domain even for an explicit treatment of convection and diffusion terms. Pressure is treated semi-implicitly with SIMPLE (Patankar 1980). Additionally, source terms arising from porous media are treated implicitly, leading to local time steps to ensure stability, i.e., for each cell in the computational grid an individual relaxation time step is applied.

Therefore, the paper is organized as follows: First, we present the transport equations for the type of problem of interest in simplified form. Emphasis is put on the modeling of the source terms arising due to the presence of porous media and their influence on pressure–velocity coupling, while the details of discretization for convection and diffusion are not considered. The developed approach leads to a non-constant system matrix for the pressure equation. Therefore, we outline a procedure for the fast recalculation of this matrix in each iteration step in the next section. Finally, we provide a 1D proof-of-concept example, and more sophisticated 2D and 3D examples.

## Theoretical Background

In this section, we review the basic equations describing the problem to be solved, and we present the necessary steps to derive the model equations.

### Governing Equations

Our main goal is to describe subsonic air flow in the presence of porous media by means of coarse grid CFD. A typical example is the air flow through a vehicle’s underhood compartment, where the different heat exchangers are modeled as porous media. Such a flow is governed by the Navier–Stokes equations

1 $$\begin{aligned} \nabla \cdot \rho \mathbf {u}= & {} 0 \end{aligned}$$

2 $$\begin{aligned} \frac{\partial \rho \mathbf {u}}{\partial t} + (\mathbf {u} \cdot \nabla )\rho \mathbf {u}= & {} -\nabla p + \mu \nabla ^2 \mathbf {u} + {\varvec{{\mathcal {S}}}}, \end{aligned}$$

where $\mathbf {u}$ and *p*, respectively, denote the independent flow-field variable velocity and pressure. The air density is given by $\rho$ and the viscosity by $\mu$. Additional body forces like the resistance of the porous media are given by the source term ${\varvec{{\mathcal {S}}}}$. It should be noted that this source terms account for geometric details which are not resolved by the numerical grid. In this approach, the flow within the porous medium is split into a free-stream part (with fully open geometry) and additional resistance forces (which account for flow area reduction and friction). Therefore, the source must be considered additionally to the convection and diffusion operators in this approach.

In vehicle aerodynamics, the heat exchanger resistance is commonly modeled by a suitable Darcy–Forchheimer law (Whitaker 1996; Rohsenow et al. 1998; Dukhan et al. 2014; Janssen et al. 2015). In our case, we follow the formulation as it is used, e.g., in OpenFOAM  (Hafsteinsson 2009), which is given by

3 $$\begin{aligned} {\mathcal {S}}_i(u_i) = {-}\left( \mu D + {\displaystyle \frac{1}{2}}\rho |u_i| F \right) u_i. \end{aligned}$$

Here, the Darcy constant *D* accounts for viscous losses (and corresponds to the inverse permeability) and the Forchheimer constant *F* for losses due to turbulent effects in the porous medium.

Generally for porous media, the sign of the source term is always opposite to the sign of the local velocity, and the derivative w.r.t. velocity is always negative, which is a crucial condition for a robust solution (Patankar 1981). Thus, an implicit treatment of the porous medium is always preferable (Merci et al. 2000).

### Discretized Equations

When developing our discretized model equations from the Navier–Stokes equations, our main goal is to obtain a steady-state solution for the momentum equation Eq. (2). In our approach, the system relaxes from some initial state toward equilibrium by applying simple forward Euler time stepping

4 $$\begin{aligned} \frac{{u_i}^{(n+1)} - {u_i}^{(n)}}{\varDelta t} = {\mathcal {C}}_i + {\mathcal {D}}_i + {\mathcal {P}}_i + {\mathcal {S}}_i, \end{aligned}$$

where *n* denotes the iteration step for a relaxation parameter $\varDelta t$ (fictitious time step). Furthermore, ${\mathcal {C}}_i$, ${\mathcal {D}}_i$ and ${\mathcal {P}}_i$ denote the discretized forms of convection, diffusion, and the pressure gradient, respectively.

To account for mass conservation, Eq. (1), we apply the SIMPLE algorithm (Patankar 1980) to establish the velocity–pressure coupling. Basically, the update procedure for velocity is then given by

5 $$\begin{aligned} {u_i}^{(n+1)} = {u_i}^{(n)} + \varDelta t \left[ {{\mathcal {C}}_i}^{(n)} + {{\mathcal {D}}_i}^{(n)} + {{\mathcal {P}}_i}^{(n+1)} + (1-\varPsi ) {{\mathcal {S}}_i}^{(n)} + \varPsi {{\mathcal {S}}_i}^{(n+1)} \right] , \end{aligned}$$

where the superscripts (*n*) and $(n+1)$ denote, for which iteration step the discretization operators should be evaluated. Please note that the implicit treatment of the pressure via ${{\mathcal {P}}_i}^{(n+1)}$ is part of the SIMPLE strategy to ensure mass conservation, while the blending factor $\varPsi$ makes the source term implicit.

For the further treatment of the model equations, we make the following assumptions, which are well fulfilled by the Darcy–Forchheimer law Eq. (3):

- The source term depends explicitly on the velocity, i.e., an analytical dependence of the source term on velocity is provided: ${\mathcal {S}}_i = {\mathcal {S}}_i(u_i)$.
- The source term is sufficiently smooth to be linearized.

By virtue of these assumptions, the implicit contribution of the source term can be written in linearized form as

6 $$\begin{aligned} {{\mathcal {S}}_i}^{(n+1)}\approx & {} {{\mathcal {S}}_i}^{(n)} + {\left( \frac{\text {d}{\mathcal {S}}_i}{\text {d}u_i}\right) }^{(n)} \left( {u_i}^{(n+1)} - {u_i}^{(n)} \right) \nonumber \\:= & {} {{\mathcal {S}}_i}^{(n)} + {{\mathcal {S}}_i^\prime }^{(n)} \left( {u_i}^{(n+1)} - {u_i}^{(n)} \right) . \end{aligned}$$

Furthermore, we consider only linear discretizations of the pressure gradient. This allows to split the implicit pressure contribution into a contribution from the previous iteration and a corrective contribution, such that

7 $$\begin{aligned} {p}^{(n+1)}:= & {} {p}^{(n)} + {\varDelta p}^{(n)} \Longrightarrow {\mathcal {P}}\left( {p}^{(n+1)}\right) = {\mathcal {P}}\left( {p}^{(n)}\right) + {\mathcal {P}}\left( {\varDelta p}^{(n)}\right) \nonumber \\:= & {} {{\mathcal {P}}}^{(n)} + {\varDelta {\mathcal {P}}}^{(n)}. \end{aligned}$$

By inserting the simplifications Eqs. (6) and (7) into the update rule Eq. (5) and introducing an intermediate velocity $u_i^*$, we obtain

8 $$\begin{aligned} \nonumber {u_i}^{(n+1)}= & {} \left\{ {u_i}^{(n)} + \varDelta t \left[ {{\mathcal {C}}_i}^{(n)} + {{\mathcal {D}}_i}^{(n)} + {{\mathcal {P}}_i}^{(n)} + {{\mathcal {S}}_i}^{(n)} + \varPsi {{\mathcal {S}}_i^\prime }^{(n)} \left( u_i^* - {u_i}^{(n)}\right) \right] \right\} \\&+\,\varDelta t \left[ {\varDelta {\mathcal {P}}_i}^{(n)} + \varPsi {{\mathcal {S}}_i^\prime }^{(n)}\left( {u_i}^{(n+1)}-u_i^* \right) \right] . \end{aligned}$$

To fix the value of the yet unknown intermediate velocity, we demand that it is equal to the term within curly braces in the above equation, yielding the final update rule

9 $$\begin{aligned}&\displaystyle \varDelta t^* := \frac{\varDelta t}{1 - \varDelta t \varPsi {{\mathcal {S}}_i^\prime }^{(n)}} \end{aligned}$$

10 $$\begin{aligned}&\displaystyle u_i^* = {u_i}^{(n)} + \varDelta t^* \left[ {{\mathcal {C}}_i}^{(n)} + {{\mathcal {D}}_i}^{(n)} + {{\mathcal {P}}_i}^{(n)} + {{\mathcal {S}}_i}^{(n)} \right] \end{aligned}$$

11 $$\begin{aligned}&\displaystyle {u_i}^{(n+1)} = u_i^* + \varDelta t^* {\varDelta {\mathcal {P}}_i}^{(n)}. \end{aligned}$$

This update procedure corresponds to that of the SIMPLE algorithm with explicit source terms, except for the position-dependent time step $\varDelta t^*$.

The obvious advantage of this approach is that the implicit treatment of the source terms neither increases the complexity nor decreases the speed of the SIMPLE algorithm, as no additional equation solving is required.

### Pressure–Velocity Coupling

To complete the procedure presented in the previous section, a suitable pressure–velocity coupling must be implemented to ensure mass conservation according to the continuity equation Eq. (1). In the discretized form, continuity is given by

12 $$\begin{aligned} {\mathcal {L}}\left( {\mathbf {u}}^{(n+1)} \right) = {\mathcal {L}}\left( \mathbf {u}^* + \varDelta t^* {\varDelta {\mathcal {P}}}^{(n)} \right) = 0, \end{aligned}$$

where the operator ${\mathcal {L}}$ denotes the discretized divergence operator. By assuming that the divergence operator ${\mathcal {L}}$ is linear, mass conservation can be written as

13 $$\begin{aligned} {\mathcal {L}}\left( \varDelta t^* {\varDelta {\mathcal {P}}}^{(n)} \right) = - {\mathcal {L}}\left( \mathbf {u}^* \right) , \end{aligned}$$

where the r.h.s. can be calculated after the predictor step from the intermediate $u^*$. By introduction of the influence function ${\mathcal {F}}$ with

14 $$\begin{aligned} {\mathcal {F}}\left( {\varDelta p}^{(n)} \right) := {\mathcal {L}}\left( \varDelta t^* {\varDelta {\mathcal {P}}}^{(n)} \right) = {\mathcal {L}}\left[ \varDelta t^* {\mathcal {P}}\left( {\varDelta p}^{(n)}\right) \right] \end{aligned}$$

and the fact that the combined application of divergence and gradient operator can also be written as a matrix operation, the mass conservation takes the final form

15 $$\begin{aligned} {\mathcal {F}}\left( {\varDelta p}^{(n)} \right) = A {\varDelta p}^{(n)} = - {\mathcal {L}}\left( \mathbf {u}^* \right) , \end{aligned}$$

where *A* is the pressure influence matrix. Contrary to the explicit case, this matrix is not constant for all iterations steps, but has to be updated in each step because of the derivative ${{\mathcal {S}}_i^\prime }^{(n)}$ which is part of the time step $\varDelta t^*$ in Eq. (12).

**Fig. 1:**
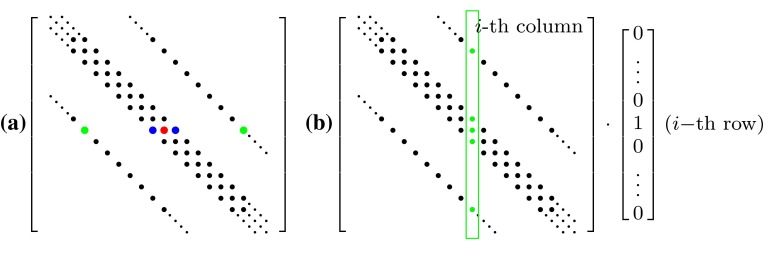
**a** Typical structure of the pressure influence matrix for a 2D case. The *red dot* indicates a cell, which contains an additional source term due to a porous medium. The original matrix is changed due to source terms in *x*-direction (*blue*), *y*-direction (*green*) and both of them (*red*). **b** Projection of *i*-th column of influence matrix by suitable test vector

For a Cartesian grid with a first-order pressure gradient, the pressure influence matrix has a predominantly diagonal structure with six (3D) and four (2D), respectively, occupied side diagonals (c.f. Fig. 1). Additional matrix elements occur locally next to these “geometry” elements due to the implicit treatment of the porous media. Our target is to place an arbitrary number of porous regions within the computational domain. Moreover, we want to keep the approach general, i.e., not only for Darcy–Forchheimer porosities Eq. (3), but for any law which describes the pressure drop in dependence of velocity sufficiently smooth and monotonic. To this end, we describe the system not directly by the influence matrix *A*, but we claim to know the different influence functions to describe geometry $({\mathcal {F}}_G)$ and porous media $({\mathcal {F}}_{P,i})$, where the latter can be derived from the pressure loss law. Due to this splitting, the total influence function reads

16 $$\begin{aligned} {\mathcal {F}}\left( {\varDelta p}^{(n)} \right) = {\mathcal {F}}_G\left( {\varDelta p}^{(n)} \right) + \sum _i {\mathcal {F}}_{P,i}\left( {\varDelta p}^{(n)} \right) . \end{aligned}$$

To determine the influence matrix automatically for an arbitrary number of influence functions, the following testing technique is applied: First, a series of pressure test vectors is defined by

17 $$\begin{aligned} \varDelta \hat{p}_\ell := \delta _{\ell m},\quad \ell ,m = 1,\dots ,M, \end{aligned}$$

where *M* is the number of unknown pressure values in general the number of cells in the numerical grid and the Kronecker delta $\delta _{\ell m}$ is defined by

18 $$\begin{aligned} \delta _{\ell m} = \left\{ \begin{array}{ll} 1 &{}\quad \text {if}\; \ell = m \\ 0 &{}\quad \text {else}. \end{array} \right. \end{aligned}$$

Then, these test vectors are subsequently applied to the operator ${\mathcal {F}}$ to give the columns of the influence matrix by virtue of Eq. (13), c.f. Fig. 1, as

19 $$\begin{aligned} {\mathcal {F}}\left( \varDelta \hat{p}_\ell \right) = A_{im} \delta _{\ell m} = A_{i \ell }. \end{aligned}$$

This method does not represent a new way of how velocity and pressure are coupled (this is still according to the SIMPLE algorithm). Many methods for this purpose have been proposed and intensively studied in the literature (Patankar 1980; Ferziger and Perić 2002; Raithby and Schneider 1979; Doormaal and Raithby 1984). The approach considered here is a formal framework, which can be applied to velocity–pressure coupling algorithms which perform a predictor step for the velocity equation and a corrector step for pressure equation to fulfill mass conservation:

1. Predictor step: $u^{(n)} \rightarrow u^*$ with mass imbalance $\varDelta m$.
2. Corrector step: $$A \varDelta p = \varDelta m$$.

The necessary influence matrix *A* is not assembled directly, but all the details about the used discretization scheme are contained in the influence operator ${\mathcal {F}}$.

In this way, an easy and straight forward determination of the influence matrix is possible for any combination of applied source terms. The price to pay is that the operator ${\mathcal {F}}$ has to be evaluated *M* times per iteration step.

### Accelerated Pressure–Velocity Coupling

To overcome the drawback mentioned in the last section, the fact that usually finite stencils are applied for the discretized evaluation of divergence ${\mathcal {L}}$ and gradient ${\mathcal {P}}$ can be exploited to reduce the number of required test vectors.

**Fig. 2:**
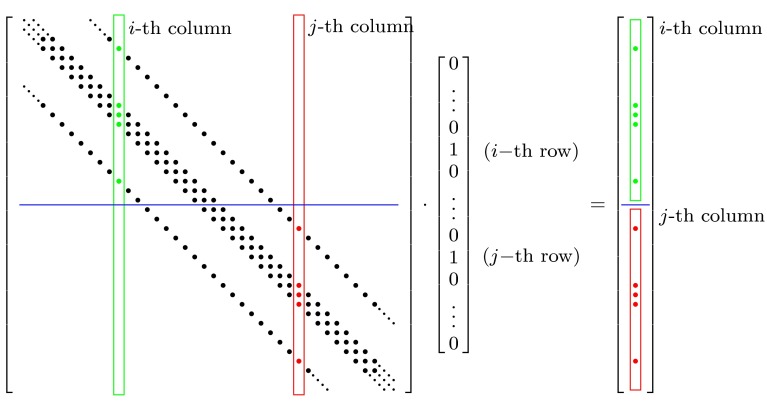
Pressure influence structure matrix $A^0$. The *i*-th (*green*, $a_i^0$) and *j*-th (*red*, $a_j^0$) column don’t have any nonzero elements in the same row, i.e., their stencils don’t overlap: $a_i^0 \cdot a_j^0 = 0$

To this end, the single columns of the pressure influence matrix will be denoted by $\mathbf {a}_j$. The “structure” of the influence matrix can be described by the vectors $\mathbf {a}_j^0$ with

20 $$\begin{aligned} A^0 = \left\{ \mathbf {a}_j^0\right\} _m = a_{j,m}^0 := 1-\left| \text {sgn}\left( a_{j,m}\right) \right| , \end{aligned}$$

where the sign function sgn is defined by

21 $$\begin{aligned} \text {sgn}\left( x\right) := \left\{ \begin{array}{lll} -1 &{}\quad \text {if} &{} x < 0 \\ 0 &{}\quad \text {if} &{} x = 0 \\ 1 &{}\quad \text {if} &{} x > 0, \end{array} \right. \end{aligned}$$

so that all non-empty matrix elements are marked by a 1, as shown in Fig. 2. As the structure of the matrix is constant during calculation, and only the values change, these vectors can be determined in a preliminary step. Then, it is possible to find sets of columns denoted by ${\mathcal {K}}_i = \kappa _{i,l}$, such that the corresponding stencils of each set are completely independent, which can be expressed by

22 $$\begin{aligned} \mathbf {a}_{l_1}^0 \cdot \mathbf {a}_{l_2}^0 = \delta _{l_1,l_2} \quad \forall (l_1, l_2) \, \in {\mathcal {K}}_i. \end{aligned}$$

These sets can be chosen such that

- $$\bigcup _i ({\mathcal {K}}_i) = \{1,\dots ,M\}$$,
- $$\cap ({\mathcal {K}}_j, {\mathcal {K}}_k) = \emptyset $$ for $$j \ne k$$,

i.e., the sets span the entire range of admissible columns without selecting any column twice.

Once these sets have been defined, new pressure test vectors can be constructed by

23 $$\begin{aligned} \varDelta p_i^* := \sum _l \varDelta \hat{p}_{\kappa _{i,l}}. \end{aligned}$$

Since the stencils in the selected columns $\kappa _{i,l}$ are completely independent, a unique mapping can be established to reconstruct the influence matrix columns $\mathbf {a}_{\kappa _{i,l}}$ from the testing operation ${\mathcal {F}}( \varDelta \hat{p}_i^* )$. Namely, all rows, for which the matrix structure vector $a_j^0, j \in {\mathcal {K}}_i$ are nonzero, belong to column *j* of the influence matrix.

The number of independent sets ${\mathcal {K}}$ depends on the used stencil for the discretization of the pressure gradient. Therefore, a constant number of test vectors $\varDelta p_i^*$ are necessary to obtain the pressure influence matrix, regardless of the number of cells in the numerical grid. For our typical application example with coarse grids with $10^3$ to $10^4$ cells, the number of test vectors is reduced to less than 20. Due to this fact, a tremendous speed-up of the calculation is possible, keeping in mind that the influence matrix must be reconstructed in every iteration step.

## Numerical Results

In the following, we present two application examples of the developed model equations to proof the concept and applicability.

### 1D Flow Through a Pipe

**Fig. 3:**
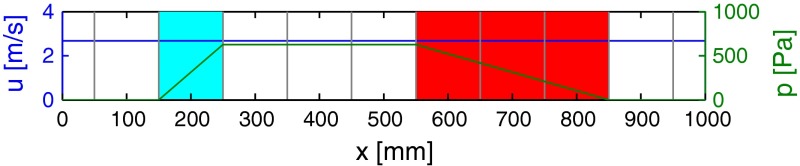
Geometry for proof-of-concept calculation including regions with constant (*cyan*) and velocity-dependent (*red*, Darcy–Forchheimer) body forces. The analytical results are indicated for velocity (*blue*) and pressure (*green*), and the coarse grid resolution is indicated by *gray lines*

As a proof-of-concept calculation, the laminar flow through a pipe is considered. On both sides of the pipe, a fixed pressure of 0 Pa is prescribed, while zero gradient conditions for velocity are applied. To drive the flow, a constant body force is given in the front region of the pipe (150 $\le$ x $\le$ 250 mm) in terms of a dimensionless Cp-value. In the rear region (550 $\le$ x $\le$ 850 mm), a porous medium with Darcy–Forchheimer law is positioned (cf. Fig. 3; Table 1).

**Table 1:** Parameters for proof-of-concept calculation

$C_p [1]$	$U_\infty$ (km/h)	$\rho$ (kg/m$^3$)	$\mu$ (Pa s)	*D* (1/m$^2$)	*F* (1/m)
0.8	130	1.2	$1.8 \times 10^{-5}$	$3 \times 10^7$	150

According to the standard definition of the pressure coefficient $(C_p)$, the pressure increase in the front region of the pipe is given by

24 $$\begin{aligned} \varDelta p = \frac{1}{2}\rho U_\infty ^2 C_p = 625.93 \,\text {Pa}. \end{aligned}$$

By rewriting the source term for the Darcy–Forchheimer law, Eq. (3), in terms of the pressure drop over the porous region thickness $L = 0.3$ m, the equilibrium flow velocity *u* is given by

25 $$\begin{aligned} \varDelta p = L \left( \mu D + \frac{1}{2}\rho F u \right) u \ \Rightarrow \ u = 2.67 \,\text {m}/\text {s}. \end{aligned}$$

To demonstrate the effect of the implicit source term treatment independently of the CFL condition, the convection term is not considered for the present example, as the flow velocity is constant due to mass conservation, anyway.

**Fig. 4:**
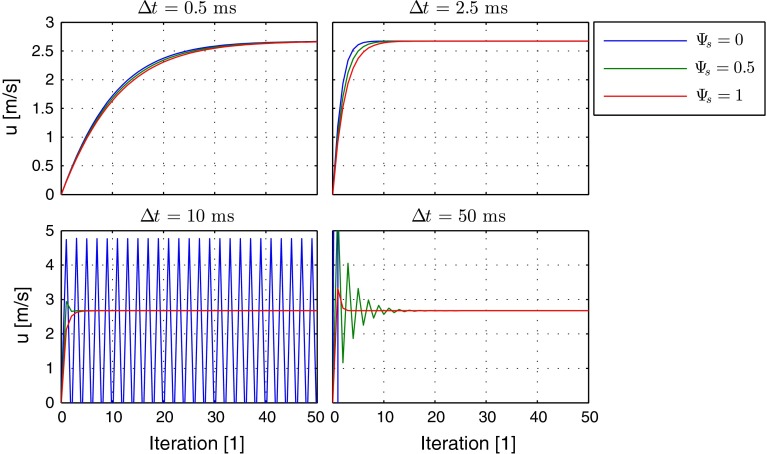
Iteration history of velocity in simple channel for different relaxation time steps for explicit (*blue*), mixed (*green*) and implicit scheme (*red*)

**Fig. 5:**
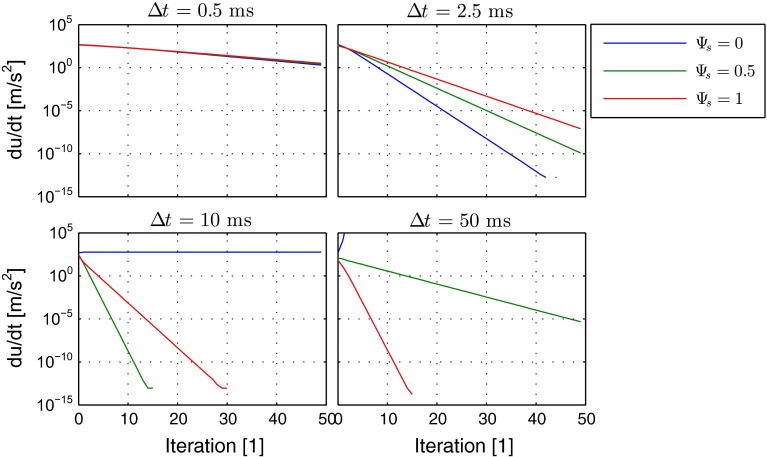
Iteration history of residuals in simple channel for different time steps for explicit (*blue*), mixed (*green*) and implicit scheme (*red*)

The iteration history for different relaxation time steps is given in Fig. 4 for the velocity and in Fig. 5 for the residual $\text {d}u/\text {d}t$. It can be observed that by application of the implicit source terms the correct solution can be obtained over a wide range of admissible relaxation time steps. A fully explicit scheme provides a correct solution only for small time steps, shows oscillatory behavior for medium size time steps, and does not converge at all for large time steps. For certain medium size time steps, a blending with factor $\varPsi _s = 0.5$ provides a faster convergence than the fully implicit treatment, but for large time steps the fully implicit treatment is superior.

Consequently, in terms of robustness of the method, a fully implicit treatment with $\varPsi _s = 1$ is the method of choice, which will be applied for the further test cases.

### 2D Flow Through a Channel

**Fig. 6:**
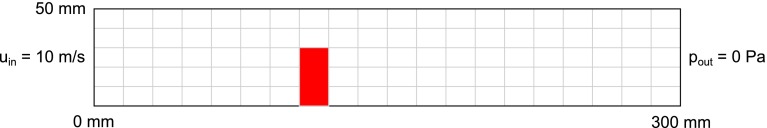
Geometry for 2D channel flow with Darcy–Forchheimer porous medium in red. The coarse grid resolution is indicated by *gray lines*

As a second example, the flow through a 2D channel partially blocked by a porous medium is considered. The problem dimensions as well as boundary conditions are shown in Fig. 6. It should be noted that a particular coarse grid is chosen for this demonstration example to investigate the application of the semi-implicit scheme in the framework of the CCGM (Langmayr et al. 2013). The porosity coefficients in longitudinal *x*-direction are given by

- Darcy coefficient $$D = 3 \times 10^7 \; [1/\text {m}^2]$$
- Forchheimer coefficient $$F = 150 \; [1/\text {m}]$$

In the present case, the porous medium should represent the strongly anisotropic character encountered, e.g., in micro-channel heat exchangers. It is common practice to model the Darcy–Forchheimer coefficients in the lateral direction, by considering an additional factor $f_\mathrm{lat}$ compared to the longitudinal direction. Usually, this factor is in the range of $10^2$–$10^4$ (Hafsteinsson 2009).

**Fig. 7:**
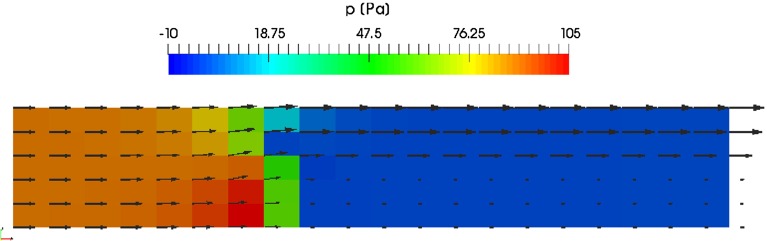
Pressure distribution in channel with velocity at cell centers. The velocity vectors at the inlet on the *left side* correspond to 10 m/s

**Fig. 8:**
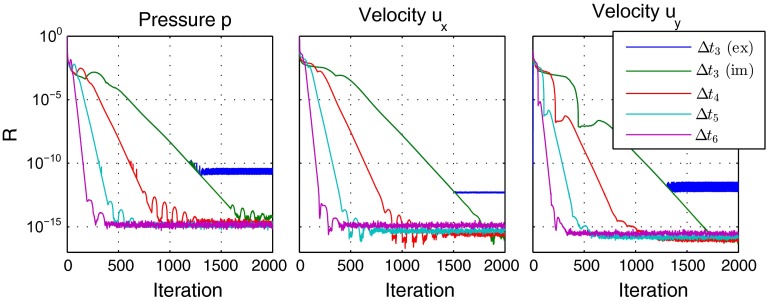
Residuals for anisotropic porous medium with $f_\mathrm{lat} = 100$ in dependency of effective relaxation step $\varDelta t_i = 2^i\varDelta t_0$. For the smallest considered time step $\varDelta t_3$ explicit (ex) as well as implicit (im) calculations were performed

The first tests are performed for the weak anisotropic case with $f_\mathrm{lat} = 100$. The pressure distribution and velocities for this case are shown in Fig. 7, with an average pressure at the inlet of $\bar{p}_{in} = 89$ Pa. Convergence tests for the explicit and implicit treatment of the source terms are conducted by performing calculations for different effective relaxation steps $\varDelta t$. The basic step size is given by $\varDelta t_0 = 10^{-4}$ s and the further steps by a series with $\varDelta t_i = \varDelta t_0 * 2^i$ s. The explicit case shows a stable convergence behavior for $\varDelta t_0$ to $\varDelta t_3$. With an implicit treatment of the source terms, step sizes up to $\varDelta t_6$ are admissible. Detailed convergence curves are shown in Fig. 8, where the residuals for iteration step *n* are defined as

26 $$\begin{aligned}&R_p(n) := \frac{\max \left( \left| p^{(n+1)} - p^{(n)}\right| \right) }{\bar{p}_ {in}} \end{aligned}$$

27 $$\begin{aligned}&R_{u_d}(n) := \frac{\max \left( \left| u_d^{(n+1)} - u_d^{(n)}\right| \right) }{u_{in}}, \quad d = x,z. \end{aligned}$$

The residuals for pressure and longitudinal velocity component show a predominantly exponential decay rate as theoretically expected. For the vertical velocity component, additional kinks are visible due to the source terms during the first iteration phase.

**Fig. 9:**
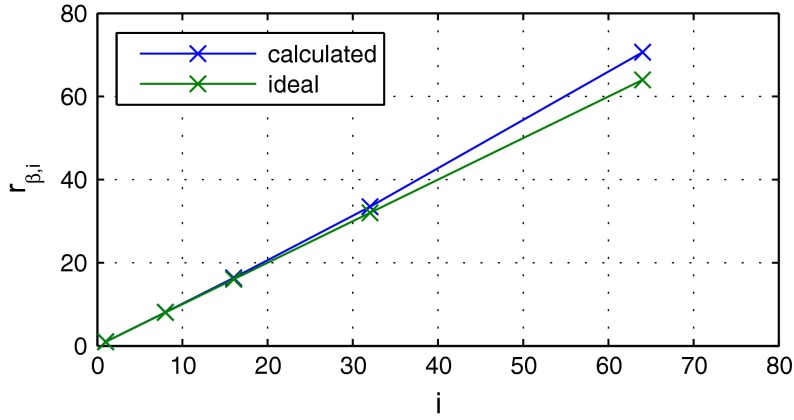
Relative decay coefficient $r_{\beta ,i}$ in dependency of the effective relative step size *i*

If the relaxation time step is small, so that also an explicit treatment is possible, the explicit and implicit scheme show the same convergence behavior in the initial iteration history, which was already observed for the 1D proof-of-concept calculation. However, the explicit scheme is not able to converge to the same precision as the implicit scheme. For example, the pressure residual displays saturation at about $R_p = 10^{-10}$ (c.f. Fig. 8).

Considering the exponential decay $R = \exp \left( -\beta _i n \right)$ with the decay rate $\beta _i := \beta \varDelta t_i$, the ratio $r_{\beta ,i} := \beta _i / \beta _1$ should correspond to $r_{\beta ,i} \equiv i$ in the case of constant system matrices. A comparison between the ideal case and the decay coefficients extracted from the residuals in Fig. 8 is shown in Fig. 9. Although in our case the system matrices are not constant due to the local time step, Eq. (9), a decay behavior as predicted is observed. For the special application example, even a better convergence than in theory is achieved.

The execution wall times for the different cases are listed in Table 2. All calculations were performed for 2000 iterations on a laptop with Intel Core i7 CPU at 2.13 GHz. The used program code was in prototype state without any optimizations performed. The time difference between explicit and semi-implicit treatment of the source terms is about 2 %. With the largest possible relaxation step with semi-implicit scheme, a speed-up of about three can be achieved compared to the largest admissible step size with an explicit scheme (not taking into account that convergence is reached much faster and less than 2000 iterations would be required). If only 750 iterations are considered, the gain in speed is more than five.

**Table 2:** Required calculation times for 2000 iterations for the different relaxation time steps

Case (relaxation time step)	$\varDelta t_3$ (ex)	$\varDelta t_3$ (im)	$\varDelta t_4$	$\varDelta t_5$	$\varDelta t_6$
Wall time (s)	95.2	97.2	64.0	51.6	34.5

**Fig. 10:**
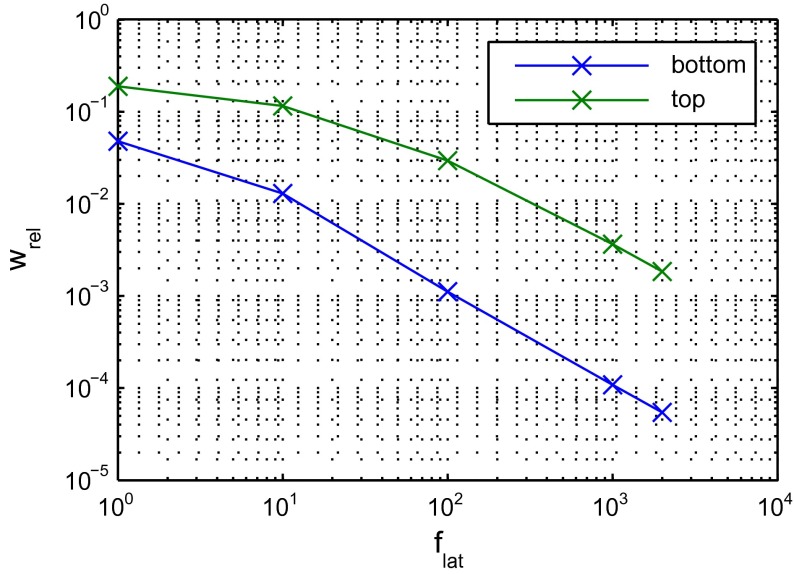
Vertical velocity relative to inlet velocity within porous medium in dependency of anisotropy parameter $f_\mathrm{lat}$

Finally, the influence of the factor $f_\mathrm{lat}$ to model anisotropy is investigated. Figure 10 shows the relative vertical velocity component $w_{rel} := w / u_{in}$ in the topmost and bottommost velocity cell within the porous region. In the ideal case, the vertical velocity component should converge to 0, as the diameter of the micro channels goes to 0. For the considered case, the relative vertical velocity is about 3 % for $f_\mathrm{lat} = 100$ and less than 1 % for $f_\mathrm{lat} = 1000$ without the need to introduce additional baffles. For this degree of anisotropy, the calculation is stable for effective step sizes as large as $\varDelta t_6$.

### 3D Flow Through a Channel

**Fig. 11:**
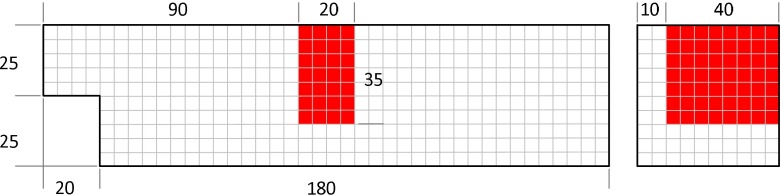
Geometry for 3D channel flow with Darcy–Forchheimer porous medium in *red*. The coarse grid resolution is indicated by *gray lines*. Length is given in millimeter

The final example treats the flow through a 3D channel partially blocked by a porous medium. The basic geometry is similar to the front region of the Pitz-Daily backward facing step (Pitz and Daily 1983). Additionally, a porous medium is placed in the upper half of the channel, c.f. Fig. 11. The boundary conditions and Darcy–Forchheimer coefficients are the same as for example 2. The factor for lateral forces was 1000 if not noted otherwise. Again, it should be noted that a particular coarse grid is chosen for this demonstration example to investigate the application of the semi-implicit scheme in the framework of the CCGM (Langmayr et al. 2013).

**Fig. 12:**
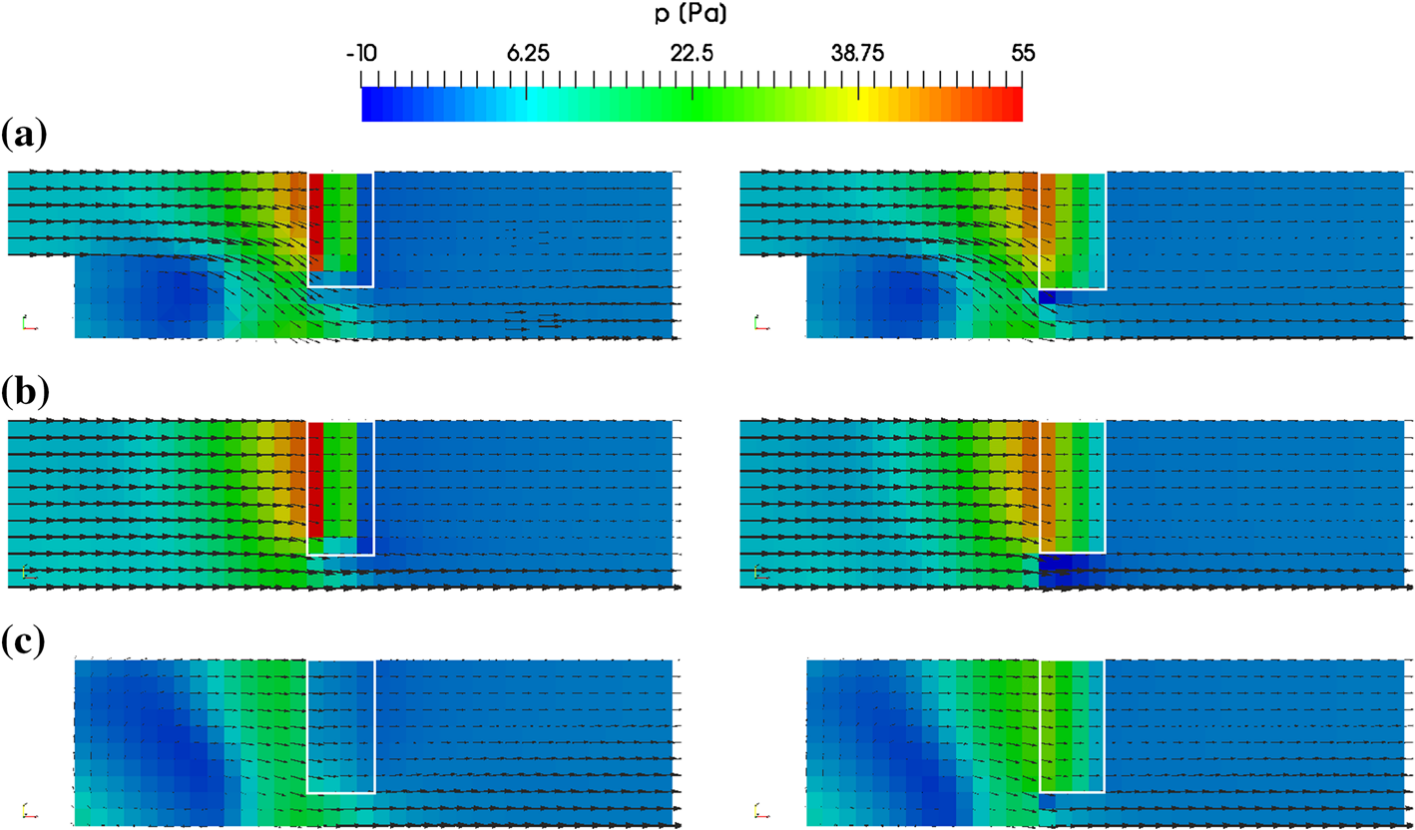
Comparison of pressure distribution calculated with OpenFOAM  (*left*) and presented method (*right*) for cut planes at *y* = 0 mm (**a**), *z* = 10 mm (**b**) and *z* = $-$10 mm (**c**). Velocity is indicated by *black arrows* which correspond to 10 m/s at the inlet on the *left side*. The position of the porous medium by the *white rectangle*

**Fig. 13:**
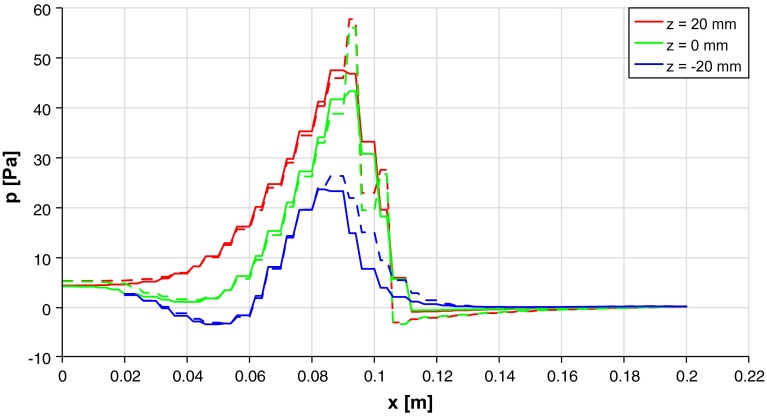
Comparison of pressure in in plane *y* = 0 at different heights *z* between current approach (*solid*) and OpenFOAM (*dashed*)

A comparison of the pressure distribution and velocities in the channel calculated with OpenFOAM and the proposed method is shown in Fig. 12 for different cut planes. Line plots of the in *x*-direction at $y = 0$ mm for different values of *z* are shown in Fig. 13. There is a good match of the results between the inlet and the entry plane of the porous medium. In the first cell row of the porous medium, OpenFOAM predicts higher pressures than our method and in the last cell row lower pressures. In the region behind the porous medium, the results obtained OpenFOAM show a small pressure increase, which seems to be unphysical at this position. The same is not the case for the proposed method.

## Conclusions

In this paper, we presented a method for a semi-implicit modeling of porous media in the steady-state Navier–Stokes equations. This method corresponds to applying a suitable local time step in porous regions in the framework of time-explicit relaxation methods. Therefore, only the pressure equation has to be solved, which allows for a fast calculation. The applicability of this method was tested by three different cases: In the first case, the flow was driven by a body force and in the other ones by external boundary conditions. Since the semi-implicit treatment allows for a much larger relaxation time step, about ten times less iterations are necessary compared to the explicit treatment, to achieve the same residuals. This corresponds to a speedup of five in terms of wall time.

Furthermore, an automatic procedure to identify the source terms was presented and applied to the test cases. This procedure allows to “test” the influence of the porous medium on the flow structure. The advantage is that in this way also porous media can be considered, for which no analytic characteristic curve is available—provided that the characteristics is sufficiently smooth and monotonic. This method to determine the pressure influence matrix runs with a constant number of test operations independently on the used grid size. Therefore, it has hardly any influence on the calculation time for usual calculation setups, where the free-stream volume is large compared to the volume occupied by the porous medium.

Due to the observed properties, the proposed semi-implicit approach is a well-suited method for accelerating calibrated coarse grid methods (CCGM). As a consequence, the semi-implicit treatment of source terms arising in the presence of porous media is a promising tool for accelerated 1D–3D coupling in vehicle underhood flow calculation.
